# Reproducibility of malaria sporozoite challenge model in humans for evaluating efficacy of vaccines and drugs: a systematic review

**DOI:** 10.1186/s12879-021-06953-4

**Published:** 2021-12-20

**Authors:** Workineh Shibeshi, Wilhelmina Bagchus, Özkan Yalkinoglu, Aliona Tappert, Ephrem Engidawork, Claude Oeuvray

**Affiliations:** 1 Department of Pharmacology and Clinical Pharmacy, School of Pharmacy, College of Health Sciences, Addis Ababa University, Addis Ababa, Ethiopia; 2 Translational Medicine, Merck Serono S.A., An Affiliate of Merck KGaA, Darmstadt, Germany; 3 Translational Medicine, Merck Healthcare KGaA, Darmstadt, Germany; 4 Global Patient Safety, Merck Healthcare KGaA, Darmstadt, Germany; 5 Global Health Institute of Merck, Ares Trading S.A., A subsidiary of Merck KGaA, Darmstadt, Germany

**Keywords:** Controlled human malaria infection, Sporozoite challenge, Malaria vaccine, Antimalarial drug, Systematic review

## Abstract

**Background:**

The development of novel malaria vaccines and antimalarial drugs is limited partly by emerging challenges to conduct field trials in malaria endemic areas, including unknown effects of existing immunity and a reported fall in malaria incidence. As a result, Controlled Human Malaria Infection (CHMI) has become an important approach for accelerated development of malarial vaccines and drugs. We conducted a systematic review of the literature to establish aggregate evidence on the reproducibility of a malaria sporozoite challenge model.

**Methods:**

A systematic review of research articles published between 1990 and 2018 on efficacy testing of malaria vaccines and drugs using sporozoite challenge and sporozoite infectivity studies was conducted using Pubmed, Scopus, Embase and Cochrane Library, ClinicalTrials.gov and Trialtrove. The inclusion criteria were randomized and non-randomized, controlled or open-label trials using *P. falciparum* or *P. vivax* sporozoite challenges. The data were extracted from articles using standardized data extraction forms and descriptive analysis was performed for evidence synthesis. The endpoints considered were infectivity, prepatent period, parasitemia and safety of sporozoite challenge.

**Results:**

Seventy CHMI trials conducted with a total of 2329 adult healthy volunteers were used for analysis. CHMI was induced by bites of mosquitoes infected with *P. falciparum* or *P. vivax* in 52 trials and by direct venous inoculation of *P. falciparum* sporozoites (PfSPZ challenge) in 18 trials. Inoculation with *P. falciparum*-infected mosquitoes produced 100% infectivity in 40 studies and the mean/median prepatent period assessed by thick blood smear (TBS) microscopy was ≤ 12 days in 24 studies. On the other hand, out of 12 infectivity studies conducted using PfSPZ challenge, 100% infection rate was reproduced in 9 studies with a mean or median prepatent period of 11 to 15.3 days as assessed by TBS and 6.8 to 12.6 days by PCR. The safety profile of *P. falciparum* and *P.vivax* CHMI was characterized by consistent features of malaria infection.

**Conclusion:**

There is ample evidence on consistency of *P. falciparum* CHMI models in terms of infectivity and safety endpoints, which supports applicability of CHMI in vaccine and drug development. PfSPZ challenge appears more feasible for African trials based on current evidence of safety and efficacy.

## Background

*Plasmodium falciparum* and *Plasmodium vivax* are the most common plasmodium species causing human malaria worldwide. Currently, RTS,S is the first and the only licensed malaria vaccine shown to provide partial protection against malaria in young children in three sub-Saharan African countries [1] The emergence of artemisinin resistance, unfavorable pharmacokinetic or toxicological profile of available drugs and other unmet medical needs highlight the need for new antimalarial drugs with novel mechanisms of action [2]. There are promising antimalarial molecules in clinical development, including KAF156 [3], KAE609 [4], DSM265 [5] and M5717, formerly DDD498 [6]. After successful completion of phase 1 safety and pharmacokinetic studies, phase 2 trials have been undertaken or are being planned in malaria-endemic areas. However, ethical and logistical problems, unknown effects of existing immunity and a reported fall in malaria incidence in endemic areas are hampering the execution of both drug and vaccine trials [2, 7]*.* As a result, Controlled Human Malaria Infection (CHMI) models are being used as emerging approaches for testing efficacy of investigational medicinal products against malaria.

CHMI entails a deliberate infection of healthy volunteers either by inoculation of plasmodium sporozoites (sporozoite challenge) by mosquito bite or direct injection of *Plasmodium falciparum* sporozoites (PfSPZ Challenge) or Plasmodium-infected erythrocytes [8]. Since the first deliberate infection of volunteers with malaria as a treatment for neurosyphilis in the 1920s, CHMI has increasingly been used to understand parasite biology and as a framework in the assessment of novel vaccine, drug, and diagnostic candidates [7, 9–11]. Indeed, CHMI studies have become a vital tool to accelerate vaccine and drug development and are integrated into First-in-Human trials. CHMI studies provide a cost-effective way to investigate efficacy and characterize the potential therapeutic dose range in early clinical development to gain exploratory proof of concept [12], and to provide an early alert for clinical safety signals.

Mosquito bite-induced CHMI has been shown to be safe and effective for efficacy testing of anti-malarial drug and vaccine candidates for more than 25 years, including early studies of subunit malaria vaccines and atovaquone efficacy [10]. There is an exponential increase in the use of CHMI models worldwide due to the availability of cryopreserved infectious *P. falciparum* sporozoites (PfSPZ Challenge), and the need to test more vaccine and drug candidates [11]. Heterogenous study designs and procedures have been used in malaria sporozoite-challenge trials that resulted in variable study outcomes. Moreover, comparative reproducibility of various modalities of sporozoite-challenge trials are lacking in the literature. The objective of this study was, therefore, to conduct a systematic review of current literature on reproducibility of efficacy, such as infection rate, prepatent period, parasitemia and safety clinical endpoints of *P. falciparum* and *P. vivax* sporozoite challenge model in humans. In addition, we evaluated the study designs, variables influencing study outcomes, optimal and standardized procedures, ethical and regulatory considerations, and limitations in the conduct of sporozoite challenge studies.

## Methods

A systematic review of the literature published between 1990 and 2018 was undertaken using public databases including Pubmed, Scopus, Embase, Cochrane Library, ClinicalTrials.gov and Trialtrove. The search was conducted from 30 September 2018 to 10 November 2018. Randomized or non-randomized and controlled or open-label trials testing efficacy of antimalaria drug and vaccine candidates conducted using *P. falciparum* or *P. vivax* sporozoite challenge through mosquito bites or needle injection and sporozoite infectivity human studies were included. Ex-vivo studies, nonclinical studies and studies based on induced blood-stage malaria using infected erythrocytes were excluded. The search was conducted using key words, including sporozoite challenge, sporozoite challenge studies, controlled human malaria infection, clinical trials, human, *P. falciparum* and *P. vivax*. Abstracts, articles and conference papers were searched without time restriction. Additionally, the grey literature was used to complement evidence synthesis. Only healthy volunteers receiving placebo but not any of the investigational vaccine or drug, and those who participated in sporozoite infectivity studies were considered for analysis of reproducibility. Infection rate, prepatent period and parasitemia were extracted as efficacy end points, while adverse events and laboratory abnormalities as safety endpoints for sporozoite challenge model. The data were extracted using standardized data extraction forms, and descriptive analysis was conducted for evidence synthesis.

## Results

**Fig. 1:**
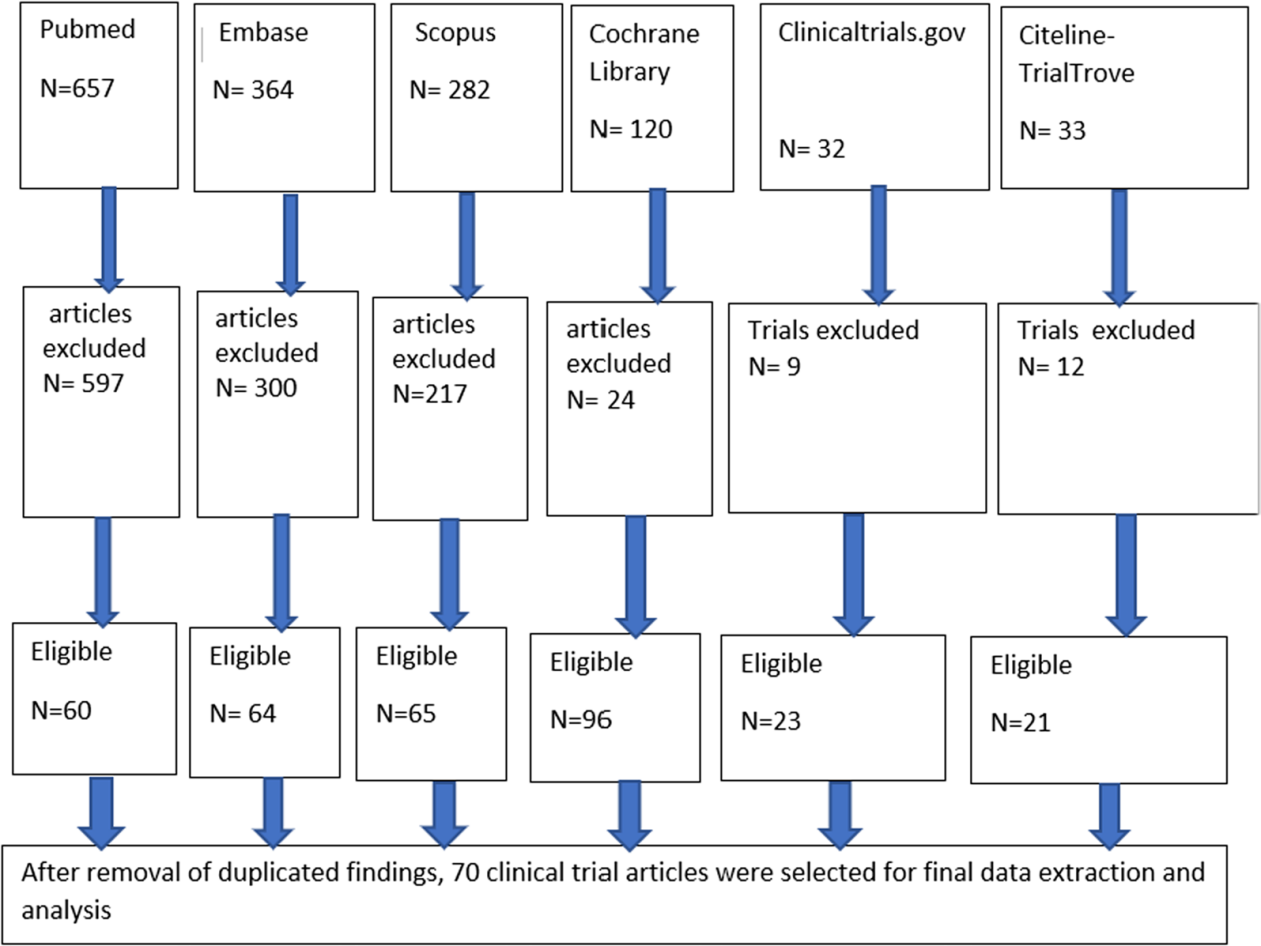
Flowchart of search results for sporozoite challenge model based studies from different databases

The procedure for screening of articles is shown in Fig. 1. We identified 1488 articles from all searched databases and 70 of them were found to be eligible, out of which 44 were vaccine trials, 19 sporozoite infectivity studies, 5 prophylactic drug trials and 2 assessments of natural immunity. The CHMI trials were conducted from 1990 to 2018 and included a total of 2329 adult healthy volunteers, out of which 1221 were on either placebo treatment used in vaccine and drug efficacy evaluation or in sporozoite infectivity trials. Table 1 summarizes the global landscape of malaria sporozoite challenge trials. The majority of *P. falciparum* challenge studies were conducted in the USA, the Netherlands, UK, Germany, and Spain; while *P. vivax* challenge studies were conducted only in Colombia and the USA. Few *P. falciparum* studies were also carried out in African countries, particularly in Tanzania, Kenya, Mali, Equatorial Guinea and Gabon.

**Table.1:** The Descriptive summary of global landscape of malaria sporozoite challenge studies conducted from 1990 to 2018

Characteristics	Number of studies
Sporozoite challenge study based on purpose	
Sporozoite infectivity studies	19
Malaria vaccine efficacy studies	44
Drug evaluation (chemoprophylaxis)	5
Acquired immunity assessment	2
Country trial conducted	
USA	31
Netherlands	11
UK	9
Germany	3
Colombia	5
Spain	2
Tanzania	2
Kenya	3
Gabon	1
Equatorial Guinea	1
Mali	2
Allocation to treatment	
Randomized placebo-controlled	39
Non-randomized controlled	16
Open-label	2
Not known	9
Masking type	
Double-blind	26
Single-blind	6
Open-label	35
Not known	3
Intervention model	
Parallel assignment	54
Single group assignment	8
Factorial assignment	2
Not known	5
Phase of study	
Phase 1	32
Phase 1/2a	20
Phase 2	6
Not applicable	6
Not known	5
Sporozoite challenge study type	
Mosquito bite based	52
PfSPZ challenge based	18
Strain/clone of plasmodium used	
PfNF54	29
Pf3D7	24
PfNF166.C8	3
PfNF135.C10	3
Pf7G8	4
Pv clinical isolate	7
Mosquito species used	
*A.stephensi*	44
*A.albimanus*	5
*A. gambiae*	1
*A.dirus*	1

Analysis of the data by method of infection indicated that 45 (64.3%) studies used *P. falciparum*-infected mosquito bites (n = 1286), 7 (10%) *P. vivax*-infected mosquito bites (n = 189), and 18 (25.7%) injection of cryopreserved *P. falciparum* sporozoites (n = 854). It is of note that only 56% (39/70) of the studies were randomized placebo-controlled trials with blinding. The sporozoite challenge studies were conducted using various *P. falciparum* strains or clones including NF54, 3D7, NF166.C8, NF135.C10, or 7G8 induced through either sporozoite inoculation or via bite of laboratory-reared mosquitoes (*A. Stephensi* or *A. gambiae).* However, only seven sporozoite challenge studies were conducted through the bite of *P.vivax* -infected mosquitoes (*A. dirus* or *A. albimanu).*

### Detection and treatment of malaria challenge infections

Following inoculation of sporozoites to healthy volunteers, parasitemia was monitored by clinical signs and symptoms, microscopic examination of thick blood smears (TBS) and DNA amplification methods (PCR). Out of the 70 studies, 53 used PCR methods with different sensitivity for parasite detection, where 18 studies used PCR, 23 qPCR and 12 RT-qPCR, but 17 studies did not use any PCR method. Rarely, thin blood smears, quantitative buffy coat and blood culture were also used for parasite detection.

In all studies, parasitemia was monitored 5 to 8 days following sporozoite inoculation until day 21 or 28. Parasitemia was treated using standard antimalarial drugs; chloroquine, atovaquone-proguanil, artemether-lumefantrine, mefloquine, or primaquine; based on sensitivity of strains of the parasite used. Subjects who continued to have negative results on blood smear from the day of sporozoite inoculation until the last date of the study (21 or 28) were prophylactically treated. The studies used different criteria for treatment initiation. Clinical signs and symptoms of malaria with confirmed TBS was used in 56 studies and detection of > 500 parasites/mL of blood with qPCR in 3 studies. In smear negative subjects, > 100–500 parasites/mL of blood with qPCR was used as criteria to initiate treatment.

### Efficacy of antimalarial drugs against sporozoite challenge

Only five studies evaluated chemoprophylactic activity of candidate antimalarial drugs against mosquito-bite- or PfSPZ challenge-induced infection. The drug molecules evaluated were DSM265 [5, 14]), atovaquone [15], atovaquone-proguanil [16] and pafuramidine [17]. A single dose of DMS265 had 100% (5/5) chemoprophylactic activity when given a day before CHMI. However, dosing 3 or 7 days before CHMI showed partial efficacy (33%, 2/6), presumably due to insufficient exposure to DMS265 at the time of sporozoite challenge.

Atovaquone was also found to be 100% chemoprophylactic when given a day before CHMI and atovaquone-proguanil was 100% protective given 4 days after CHMI. Pafuramidine administration 1 or 8 days before sporozoite challenge was not effective, as it conferred protection to 6.3% (1/16) of the participants.

### Efficacy of malarial candidate vaccines against sporozoite challenge

This review indicated that sporozoite challenge has been largely used for evaluating efficacy of vaccine candidates against *P. falciparum* infection, mainly induced through bite of infected mosquitoes. The outcome of efficacy studies of vaccines is summarized in Table 2. Immunization with irradiated mosquito bite showed over 90% efficacy in *P. falciparum* [18] and 42% in *P. vivax* infections [19]. PfNF54 immunization, with mosquito bite under chloroquine prophylaxis (NF54 -CPS), showed up to 100% protective efficacy against homologous PfNF54 and Pf3D7 strains [20–24]. This approach of immunization was, however, less effective for heterologous strains [22, 25]. The protective efficacy of chemo-attenuated PfSPZ challenge (CVac) was 33–100% against PfSPZ challenge [26], while that of irradiated PfSPZ vaccines was 20–100% against PfSPZ challenge [27] and 66–100% against mosquito-bite including heterologous strains [28, 29].

**Table.2:** The protective efficacy of various types of sporozoite-based malaria vaccines against homologous and heterologous sporozoite—induced malaria infections

Vaccine category	Vaccination delivery	CHMI-strain	Vaccine efficacy (%,n)	References
NF54 -CPS	NF54-infected mosquitoes	PfNF54	100(10/10)	[20]
NF54 -CPS	NF54-infected mosquitoes	Pf3D7	100 (5/5)	[21]
NF54 -CPS	NF54-infected mosquitoes	NF54, NF166.C8, NF135.C10	100(5/5)-for NF54 20(2/10)-forNF135.C10 11(1/9)- for NF166.C8	[22]
NF54 -CPS	NF54-infected mosquitoes	NF135.C10	15(2/13)	[25]
NF54 -CPS	NF54-infected mosquitoes	PfNF54	70(7/10)	[23]
NF54 -CPS	NF54-infected mosquitoes	PfNF54	66(4/6)	[24]
NF54-irradiated	NF54-infected mosquitoes	NF54, Pf3D7, Pf7G8	92(24/26)	[18]
NF54-irradiated	Infected mosquito	PfNF54, 3D7	33(1/3)	[33]
X-irradiated Pv-SPZ	Infected mosquito	Pv	42(5/12)	[19]
RTS, S /AS01B, RTS,S/AS02A	IM injection	Pf3D7	32–50	[32]
RTS,S	IM injection	Pf3D7	41(9/22)	[34]
RTS,S	IM injection	Pf3D7	29(2/7)	[35]
RTS,S/SBAS2	IM injection	Pf3D7	43(18/41)	[36]
RTS,S/AS02A	IM injection	Pf3D7	42(8/19)	[31]
Ad35.CS.01- RTS,S/AS01	IM injection	Pf3D7	44 and 52	[37]
RTS,S/AS02A and MVA-CS	IM/ID injection	Pf3D7	33(4/12)	[38]
RTS,S	IM injection	Pf7G8	14(2/14)	[39]
RTS,S/AS01B with ME-TRAP	IM injection	Pf3D7	82(14/17) & 75(12/16)	[40]
RTS,S/AS01	IM injection	Pf3D7	63(10/16) & 87(26/30)	[41]
R32N51-81	IM injection	Pf3D7	36(4/11)	[42]
ChAd63-MVA CS, & ChAd63-MVA ME-TRAP	IM injection	Pf3D7	7(1/15) &13(2/15)	[43]
PfSPZ vaccine	IV injection	PfNF54, 3D7	66(6/9) &100(6/6)	[28]
PfSPZ vaccine	IV injection	NF54, NF166.C8, NF135.C10	92(12/13)-homologous 80(4/5)-heterologous	[29]
PfSPZ challenge	DVI	PfSPZchallenge injection by DVI	33(3/9), 100(9/9)	[26]
PfSPZ vaccine	IM injection	PfSPZ challenge injection by DVI	20(4/20) & 100(4/4)	[27]

*CPS* Chemoprophylaxis

### Infectivity of malaria sporozoite challenge in healthy volunteers

The analysis of reproducibility of infectivity parameters for sporozoite challenge was done in subjects who received sporozoite challenge as infectivity controls, implemented as part of placebo-controlled vaccine or antimalarial compound efficacy trials. The subjects enrolled in the experimental sporozoite infectivity studies did not receive any investigational compound. Once infectivity of the experimental challenge (parasitemia) was confirmed, all participants had received mandatory rescue medication with confirmation of cure. Table 3 depicts a summary of the efficacy endpoints measured by different methods. A sample size of usually 6 subjects per group (range 2–18) was used in sporozoite challenge studies. The *P. falciparum* infected mosquito challenge was 100% infective in 88.9% (40/45) of the studies. In 9 studies, the infectivity was, however, came down to 88.3%, whereby one subject per group did not develop parasitemia [9, 19, 24, 28, 36, 44–46].

**Table.3:** The infectivity, prepatent period and parasitemia measurements in healthy volunteers in malaria sporozoite challenge participated as controls in vaccine or drug trials and in infectivity studies

Plasmodium spp/strain	CHMI type	Infectivity % (n)	TBS-Prepatent period (mean or median) days	PCR-Prepatent period (mean, median) days	PCR-parasitemia (n/µl) Mean or median	References
PfNF54	PfSPZ challenge	100(4/4)	11.7*	7.8	19.55–28.756	[5]
PfNF54	PfSPZ challenge	100(13/13)	11**			[26]
PfNF54	PfSPZ challenge	100 (5/5)	12.7*	10.3	25.6	[71]
PfNF54	PfSPZ challenge	100(6/6)	11.4/12.2*	6.8		[69]
PfNF54	PfSPZ challenge	84(5/6)	13/12.7/13*	10.6/10.3/ 9.9	0.07/0.2	[46]
PfNF54	PfSPZ challenge	100 (28/28)	12.6/11.8/12/ 16.4/13.3/11.9 **			[67]
PfNF54	PfSPZ challenge	100(18/18)	12.5/13/12**		0.5	[27]
PfNF54	PfSPZ challenge	92/91	15.4/13.5**	12.6 /11.1	0.11/0.16	[9]
PfNF54	PfSPZ challenge	100(6/6)	12.7**			[66]
PfNF54	Mosquito bite/PfSPZ	100(6/6)		8.3–9.4**	≥ 0.25	[14]
PfNF54	PfSPZ challenge	100(9/9)	11.2*			[68]
PfNF54	PfSPZ challenge	64%(7/11) 56(5/9)	16.9/19.1*			[72]
PfNF54	Mosquito bite	100(5/5)	13.5**	7		[54]
Pf3D7	Mosquito bite	100(12/12)				[37]
PfNF54	Mosquito bite	100(6/6)	11.2*	7.5	33.712	[53]
Pf	Mosquito bite	100(15/15)	11.34/ 12.54*	7/6.38	23.92/35.74	[13]
PfNF54	Mosquito bite	100(13/13)	11.2*	–	–	[78]
PfNF54	Mosquito bite	94.4(17/18)	10.9*	7.5		[45]
PfNF54	Mosquito bite	100(19/19)	10.9*	7.5	1.7	[52]
PfNF54	Mosquito bite	100(6/6)	9**	6.7		[30]
Pf3D7	Mosquito bite	100(6/6)	9–13			[79]
PfNF54	Mosquito bite	100(4/4)	9–18			[15]
PfNF54	Mosquito bite	100(4/4)	8.5**	6.3	> 0.035	[23]
Pf3D7	Mosquito bite	100(6/6)	9–13		2.7	[16]
Pf3D7	Mosquito bite	100(6/6)	12.8*			[80]
PfNF54	Mosquito bite	100(5/5)	9.2*			[20]
PfNF54	Mosquito bite	100(4/4)	12.5 *	10.8		[17]
Pf3D7	Mosquito bite	100(6/6)	12.3*			[31]
Pf3D7	Mosquito bite	100	10.7			[38]
Pf3D7	Mosquito bite	100(5/5)	11*		> 1	[56]
Pf3D7	Mosquito bite	100(6/6)	11.6 *	10.8	> 1	[57]
PfNF54	Mosquito bite	80 (4/5)	7–12			[24]
Pf3D7	Mosquito bite	100 (6/6)	12.9*			[81]
Pf3D7	Mosquito bite	100(5/5)	11.8 *			[55]
Pf3D7	Mosquito bite	100% (6/6)	11–13*			[34]
Pf3D7	Mosquito bite	95(22/23)	12			[36]
Pf3D7	Mosquito bite	100(5/5)	12.3*	9		[21]
Pf3D7	Mosquito bite	100(6/6)	9–12*	7		[51]
Pf	Mosquito bite	100(6/6)	11.8*	11.1	> 1	[58]
Pf3D7	Mosquito bite	100(6/6)	10.8			[35]
Pf3D7	Mosquito bite	100(16/16)		–	> 0.5	[82]
Pf3D7	Mosquito bite	100(11/11)	12.25 **	7.4	> 0.5	[41]
Pf3D7/7G8	Mosquito bite	100(4/4)	10–12			[39]
Pf3D7	Mosquito bite	100(36/36)	11–14			[18]
Pf3D7	Mosquito bite	100(11/11)	11–14			[42]
Pf3D7	Mosquito bite	100 (13/13)	13*			[41]
PfNF54/3D7	Mosquito bite	100 (5/5)	11–14			[33]
Pf7G8	Mosquito bite	100(4/4)	9–12			[49]
Pf	Mosquito bite	100(36/36)	10.8/11.8*			[32]
Pf3D7	Mosquito bite	100(6/6)	10.3**	7.5	> 0.5	[67]
Pf	Mosquito bite	91(11/12)	7–13			[28]
Pf3D7/Pf7G8	Mosquito bite	100(22/22)	10.9/11.6/11.9**			[29]
PfNF54/NF166.C8/ NF135.C10	Mosquito bite	100(23/23)	10.2/7.2/7.4*	7.5/6.5/6.5		[47]
PfNF135.C10	Mosquito bite	100 (5/5/)	8.5**	-	> 0.05	[25]
PfNF54/NF166.C8/ NF135.C10	Mosquito bite	100(15/15)	-	-	> 0.1	[22]
NF166.C8/ NF135.C10	Mosquito bite	100(4/4/)	7.5**	-	> 0.5	[48]
Pv	Mosquito bite	100(17/17)	12 *	-		[62]
Pv	Mosquito bite	94(15/16)	12.5/12.8 *	9.2–9.4	17.5	[64]
Pv	Mosquito bite	100(2/2)	12–13*	8–11	12.5–1050	[19]
Pv	Mosquito bite	100	11–13	–	–	[83]
Pv	Mosquito bite	94.4(17/18)	11*	9–13		[44]
PV	Mosquito bite	100(6/6)	10.7**			[65]
PV	Mosquito bite	100(18/18)	11, 11, 9 *			[63]

*Geometric mean, **Median

The infection outcomes of sporozoite challenge studies conducted through either bite of plasmodium- infected mosquito or parenteral injection of purified *P. falciparum* sporozoites are described below.

### Mosquito-based *P. falciparum* challenge studies

Our review indicates that mosquito-based *P. falciparum* sporozoite challenge studies were conducted in well-established research centers, including Walter Reed Army Institute of Research (WRAIR) and Naval Medical Research Center, Sanaria Inc (Biotechnology company), University of Maryland (USA), Seattle Biomedical (USA), Oxford University (UK), and Radboud University Medical Centre (Netherlands). The main strains used were NF54 and 3D7, while the mosquito infection rates and sporozoite load in salivary glands of mosquitoes varies from 40 to 100%.

Malaria infections were induced by 1–13 bites of laboratory-reared *P. falciparum*–infected female anopheles’ mosquitoes. Five mosquito bites were used by 39 out of 52 studies (75%) as standard challenge dose, which reproducibly resulted in optimal (100%) infection rates in healthy volunteers challenged with parasite clones such as NF54, 3D7, NF135.C10 and NF166.C8 and 7G8 [22, 25, 29, 39, 47–49]*.*

The review indicated that 100% infectivity of *P. falciparum*-infected mosquitoes was reproduced in 88.9% (41/45) of the studies (Table 3). The mean or median prepatent period for *P. falciparum* infection with TBS was ≤ 12 days in 24 studies, and between 7 and 13 days in all studies. Similarly, the mean prepatent period with qPCR was between 6 and 11 days in 9 studies. PCR detected parasites, on average, 2–6 days earlier than TBS [12, 13, 17, 21, 23, 30, 40, 44, 46, 52–54].

The studies reported variable average parasite growth rates of 8–16.8 fold per 48 h [13, 21, 51, 55] while the qPCR detected parasite densities were ranging from 0.2 to 33.7 parasites/µl of blood [13, 16, 51]. The parasite detection limits of PCR ranged from 35 to 1000 parasites/mL of blood [12, 14, 22, 23, 25, 40, 49, 56–58]. Most CHMI studies resulted in 100% infectivity through standardized five bites from *P. falciparum* infected mosquitoes. CHMI conducted using three aseptically reared mosquitoes, produced by Sanaria Inc under current GMP, however, resulted in 100% (25/25) infectivity with prepatent period of 9–12 days and fewer adverse events compared to the standard CHMI by mosquito bite [45, 52].

### CHMI with *P. vivax*-infected mosquitoes

Methods for *P. vivax* CHMI were also developed at WRAIR (USA) in collaboration with institutions in Thailand and Colombia. The *P. vivax* CHMI studies were conducted through the use of infected *A. albimaus* or *A. dirus* mosquitoes from the malaria endemic area in Colombia or Thailand. *P. vivax* sporozoite challenge studies were conducted on Duffy ( +) subjects and Duffy (−) were used as controls. Our review (Table 3) indicated that 100% infectivity of *P. vivax*-infected mosquitoes was reproduced in five studies and infectivity was 94% in one study [44]. In three of the studies, 100% infectivity was achieved through 2–4 mosquito bites [19, 62, 63]. There was no difference in rate of infection and prepatent period when different batches of infected mosquitoes were used to infect healthy volunteers [63]. The mean or median prepatent period detected by microscopy was 9–13 days [19, 44, 62, 64, 65]. The PCR detected mean prepatent time ranged 8–13 days.

The safety review of *P. vivax* CMHI indicates mild to moderate local reactogenicity due to mosquito bites. Signs and symptoms typical of uncomplicated malaria were observed but serious adverse events were not reported. Most laboratory abnormalities were associated with mild to moderate increase in liver transaminases, alkaline phosphatase and leukopenia.

### PfSPZ challenge-mediated CHMI studies

Aseptic, purified, cryopreserved *P. falciparum* sporozoites (PfSPZ challenge) was developed from NF54 strain of *P. falciparum* by Sanaria Inc in Maryland, USA. PfSPZ challenge is fully infectious and stable over time when stored in liquid nitrogen vapor phase (LNVP) below a temperature of -150 °C. To establish optimal infectivity and safety of PfSPZ challenge, several CHMI studies were conducted in healthy volunteers using different doses, injection volumes and routes of administration. These clinical studies were conducted at Radboud University Medical Center [46], the University of Oxford [66], Tanzanian Ifakara Health Institute [9], University of Maryland [45], Kenya Medical Research Institute [12, 67], University of Tübingen [68] and Barcelona Centre for International Health [69]. These studies successfully defined the reproducible regimen for administration of PfSPZ challenge resulting in 100% infection rate, with a prepatent period of ≤ 12 days and enabled replication of outcomes from mosquito-induced CHMI. Additional quality control studies to assess the reproducibility and parameters affecting the prepatent period and percentage of infectivity have been conducted and established a dose of 3,200 PfSPZ as an optimal dose [46, 59, 68, 70].

The ultimate goal of using sporozoite-based malaria vaccines such as chemo-attenuated PfSPZ challenge and irradiated PfSPZ vaccine is to vaccinate population in endemic countries and induce protective immunity. In that regard, several studies have reported optimization of PfSPZ Challenge injections given Intra-muscularly (IM) and intra-dermally (ID) [9, 46, 71]. Additional studies were conducted in low, moderate, and high exposure to *P. falciparum* population to assess the impact of naturally acquired immunity on prepatent period and infectivity rate of malaria induced by PfSPZ challenge [67, 72]. For example, the Kenyan study explored the unknown effects of prior exposure to malaria on CHMI using IM PfSPZ Challenge at doses of 25,000, 75,000 and 125,000. This study screened participants’ prior exposure to *P. falciparum* using anti-schizont and anti-merozoite surface protein 2 (MSP2) antibody assay. The study included 14 volunteers with minimum exposure (antibody negative) and 14 participants with definite exposure (antibody positive) to *P. falciparum*. The results indicated that all participants developed malaria infection with the exception of one volunteer who remained blood film negative, but qPCR positive. This volunteer had reduced parasite multiplication rate, but had the highest anti-schizont and MSP2 antibody. There was no significant difference in adverse events between minimally exposed and definitely exposed subjects. Moreover, the safety profile was similar to that reported in malaria-naïve subjects, with the exception that Kenyan participants experienced adverse events of longer duration.

Similarly, another study [72] used CHMI to investigate infection rates, parasite kinetics, and malaria symptoms in lifelong malaria–exposed (semi-immune) Gabonese adults with and without sickle cell trait. Eleven semi-immune Gabonese with normal hemoglobin, nine with sickle cell trait, and five nonimmune European controls with normal hemoglobin received 3,200 PfSPZ by direct venous inoculation (DVI). Malaria infection rates detected by qPCR were 82% (9/11), 78% (7/9), and 100% (5/5), respectively. All lifelong malaria-exposed adults controlled parasite multiplication. Adverse events were more severe in non-immune volunteers, but no other differences between the study groups were found.

The studies indicated that the minimum infectious PfSPZ challenge dose for 100% infection rate in malaria naïve healthy volunteers, with prepatent period comparable to five mosquito bites, is 3200 Pf sporozoites inoculated DVI [27, 68], 75,000 IM [69], or 50,000 ID [71]. Nevertheless, it should be noted that a 3200 sporozoite dose given by DVI was not optimal in semi-immune African population [72], although this was reported in few studies. After standardization, DVI of 3200 PfSPZ has been implemented for testing of PfSPZ vaccine efficacy in Tanzania [27], Mali [73], Germany [26], and for efficacy testing of antimalarial drugs [5, 14]. Additional similar studies in Africa are ongoing at Mali [74, 75], Equatorial Guinea [76], and Kenya [77]. The details of outcomes of PfSPZ challenge studies i.e. infection rate, prepatent period and parasitemia are presented in Table 3.

### Safety and tolerability of CHMI by Sporozoite challenge

Analysis of the safety profiles of PfSPZ challenge from all studies reviewed indicates that the safety findings included the local and systemic signs and symptoms and laboratory safety signals. The safety profiles following *P. falciparum* infections by mosquito bites indicated consistent adverse events related to mosquito bite, clinical malaria and its treatment.

Intradermal injection of PfSPZ challenge was generally safe and well tolerated, only with mild to moderate local reactogenicity such as pruritus, erythema, swelling, and systemic malaria symptoms [9, 71]. Laboratory abnormalities, such as elevations in ALT/AST, thrombocytopenia and leukopenia, were infrequent and self-limited with similar incidence rates among all subjects and normalized following treatment with antimalarial drugs and at the end of follow up. Roestenberg et al*.* [46] reported an incidence of Grade 3 adverse events (AEs) in 44% of the subjects without clinically significant laboratory abnormalities before initiation of anti-malarial treatment.

In another study [66], frequencies and severities of laboratory abnormalities during malaria infection were reported to be in concordance with those expected following *P. falciparum* infection. Similarly, IV inoculation of PfSPZ Challenge was generally well tolerated with no local solicited AEs. However, three systemic solicited Grade1AEs [27] during IV and IM administration as well as Grade 2 headache, fever, fatigue; and Grade 3 lymphopenia and neutropenia, laboratory abnormalities expected of malaria were recorded, with no serious adverse outcomes [69]. Lell et al. [72] showed that DVI of PfSPZ Challenge in semi-immune African population was well tolerated, without SAE and clinically significant laboratory abnormalities. Generally, ID, IM, and IV inoculation of PfSPZ challenge in healthy malaria naïve and semi-immune subjects was safe and well tolerated, and SAEs were very limited. The mild to moderate AEs and malaria signs and symptoms as well as laboratory abnormalities consistently observed were expected of *P. falciparum* infection and treatment, and usually normalized after treatment.

## Discussion

Our systematic review attempted to establish evidence on the reproducibility of *P. falciparum* and *P. vivax* malaria sporozoite challenge model using search of literature from several databases. Seventy studies were identified and used for analysis of data reported. The efficacy endpoints of the sporozoite challenge model were infectivity, prepatent period and parasitemia; while the safety endpoints were adverse events and laboratory abnormalities.

The majority of *P. falciparum* and *P. vivax* challenge studies were conducted in developed countries (USA, the Netherlands, UK, Germany, and Spain) while only few *P. falciparum* challenge studies were also conducted in African countries. The lack of phase 1 clinical trial centers and mosquito laboratory facilities were reasons for the small number of CHMI trials conducted in Africa [9].

Analysis of detection of malaria challenge infections in healthy volunteers indicated that following inoculation of sporozoites, parasitemia was monitored by clinical signs and symptoms, microscopic examination of thick blood smears and PCR techniques. The use of various methodologies with different sensitivity for parasite detection was a source of variability in assessing clinical endpoints. The sensitivity of thick blood film is reported to be 1–10 parasites/µl, which is roughly one-tenth of qPCR [2]. Using qPCR in sporozoite-initiated CHMI has an advantage of shortening the duration of parasitemia due to lower absolute number of parasites so as to avoid or at least reduce the appearance of clinical symptoms in participants [8, 13].

Few studies evaluated chemoprophylactic activity of candidate antimalarial molecules against mosquito-bite- or PfSPZ challenge-induced infections. The studies [5, 14–17] indicate that the variability in chemoprophylactic activity of candidate molecules was observed due to differences in dosing regimen, time of administration, pharmacokinetics and pharmacodynamics of the compounds. Nevertheless, the results reaffirm the use of the sporozoite challenge model for evaluating prophylactic activity of new chemical entities, though work related to standardization remains to be done.

The review of available evidence indicated that sporozoite challenge model has been used to assess the efficacy of several vaccine candidates. The level of protective efficacy of sporozoite-based vaccines varied depending on the type of vaccine-adjuvant system, vaccination regimen, previous exposure status, and type of strain. This model showed positive infectivity in the control arm receiving placebo, although a wide range of protective responses were observed with the different antigens. The reasons for high variability observed in the protective response are largely unknown. Some of them could be directly related to the nature of the sporozoite challenge model, such as the high intensity of the challenge compared to natural infection [30], lack of genetic diversity coverage, and sub-optimal vaccination regimens during the experimental phase. The CHMI model demonstrated its ability to predict efficacy of malaria vaccines in the field as indicated by development of the RTS, S vaccine [7, 31, 32]. However, so far, only limited vaccine candidates have been tested in population at risk of malaria infection. The translation of the results obtained in the CHMI to malaria exposed patients thus remains to be confirmed.

The mosquito-based *P. falciparum* sporozoite challenge studies were also conducted in well-established research centers in developed nations using parasite clones such as NF54, 3D7, NF135.C10 and NF166.C8. The analysis of efficacy (infectivity) of malaria sporozoite challenge in healthy volunteers shows that *P. falciparum* infected mosquito challenge was 100% infective in 88.9% of the studies with five mosquito bites resulting in 100% reproducible infections. Although infectivity exhibited a decreasing trend in some studies, the data collectively indicate very good performance of the CHMI experiments. In 84.6% (44/52) of the studies, A. stephensi mosquitoes, a major vector for malaria infections in urban areas and established in laboratories [50, 51], were used for infection with *P. falciparum*. The outcomes of these studies indicated that optimum infection could be obtained with standardized five bites of *P. falciparum* infected A. stephensi mosquitoes, regardless of the geographic origin of the infecting parasite, strongly justifying the use of CHMI in testing efficacy of drug and vaccine candidates.

The mean or median prepatent period for *P. falciparum* infection with TBS was ≤ 12 days in 24 studies, and between 7 and 13 days in all studies. Similarly, the mean prepatent period with qPCR was between 6 and 11 days in 9 studies. PCR detected parasites, on average; 2–6 days earlier than TBS. The parasite detection limits of PCRs ranged from 35 to 100 parasites/mL of blood and average parasite growth rates were also variable. This variability in PCR-detection sensitivity may influence the comparability of study outcomes across trial sites and study designs, justifying the need for standardization of PCR procedures.

The CHMI studies resulted in 100% infectivity through standardized five bites from *P. falciparum* infected mosquitoes, however, using three aseptically reared mosquitoes also resulted in 100% infectivity. This observation indicates the possibility of setting aseptic model as a new standard for CHMI trials in non-endemic areas with the advantage of reducing adverse events.

Malaria infection with bite of mosquitoes, where the probability of infection increases with the number of infectious bites, reliably reflects the natural infection process. The studies indicated that the pre-patent period depends on strain of parasite, biting dose and number of mosquitoes used. Even though five mosquito bites consistently produced 100% infectivity, impact of the number of sporozoites inoculated in those bites was not clear until recently. Statistical analysis of > 1000 experimental infections by Churcher et al. [59] indicated that the probability of infection and pre-patent period depends on the number of residual-sporozoites in salivary glands of mosquitoes. The salivary gland residual-sporozoite load per fed mosquito was scored as 0 (no sporozoites), 1 (1–10), 2 (11–100), 3 (101–1000), 4 (> 1000) [60]. However, the number of sporozoites injected into each participant in mosquito bite-initiated CHMI was highly variable. As a result, the number of sporozoites counted in mosquito salivary glands is a poor predictor of the number of sporozoites injected [7].

Few studies on CHMI with *P. vivax*-infected mosquitoes were conducted on Duffy ( +) subjects. The Duffy antigen, a glycosylated membrane protein located on the surface of red blood cells and functioning as a multi-specific receptor for several chemokines, is also the obligate trans-membrane receptor for *P. vivax* invasion of red blood cells. The low endemicity of *P. vivax* infection in sub-Saharan Africa is perceived to be due to high prevalence of Duffy (−) phenotype, which confers resistance to *P. vivax* infection. However, evidence emerging from *P. vivax* infections confirmed by molecular diagnostic tests in Duffy (−) hosts indicates an additional Duffy-independent -mechanism of transmission across sub-Saharan Africa [61].

There was similar infection outcomes in CHMI with *P. vivax* with same five mosquito bites however, *P. vivax* infected mosquitoes appear more potent compared to *P. falciparum* infection, which requires five bites for reliable infection, although this needs to be validated with more studies.

The standardization of *P. vivax* challenge is proven to be more difficult, due to unavailability of long-term in-vitro culture, however, an alternative approach has been using fresh gametocytes from infected patients to infect mosquitoes. The *P. vivax* sporozoites were generated by feeding gametocyte-infected blood to laboratory-reared mosquitoes from residents areas in Colombia and Thailand [7, 8]) or by shipping infected mosquitoes to USA for challenge study [65]. The use of fresh *P. vivax* isolates may have limitations including variability in drug sensitivity, parasite multiplication rates and prepatent periods [8]. Potential hypnozoite formation and infection relapse are additional complications from *P. vivax* sporozoite-initiated CHMI studies. In addition, screening for CYP mutations associated with increased metabolism of primaquine is required for CHMI studies involving *P. vivax* to provide a terminal treatment of liver stages of *P. vivax* infection. Generally, the current methods for sporozoite challenge through bite of *P. vivax*-infected mosquitoes is validated for its infectivity and safety in small number of studies. Hence, more studies may be needed for procedures optimization.

Review of PfSPZ challenge-mediated CHMI studies indicated that this method is well established and standardized. The studies have successfully defined the reproducible regimen for administration of PfSPZ challenge resulting in 100% infection rate, with a prepatent period of ≤ 12 days which is 3200 *P.falciparum* sporozoites given by direct venous inoculation. However, natural adaptive immunity to malaria significantly prolonged the time to parasitemia. Sickle cell trait seemed to prolong it further, whereas 20% (4/20) semi-immunes demonstrated sterile protective immunity. The observed differences in infection outcomes following PfSPZ Challenge injection in Kenyan [67] and Gabonese [72] population could be attributed to population differences in malaria exposure and induction of natural adaptive immunity. However, due to small sample size, more studies in malaria endemic populations are needed to confirm infectivity of PfSPZ challenge in malaria exposed individuals in support of the future use of PfSPZ challenge for testing efficacy of vaccines and anti-malaria drugs.

Analysis of the safety and tolerability of CHMI by Sporozoite challenge indicates local and systemic signs and symptoms and laboratory safety signals that are features of malaria infection which normalize following treatment with antimalarial drugs. The safety profiles following *P. falciparum* infections by mosquito bites indicated consistent adverse events related to mosquito bite, clinical malaria and its treatment but serious adverse events were rare. Two previous studies [84, 85] reported cardiac-related serious AEs (SAEs) (acute coronary syndrome/myocarditis), whose causality was not definitely established, following a mosquito-bite CHMI study with *P. falciparum infection*. As a result, CHMI studies recommend assessment of markers for cardiac damage and coagulation such as troponin, lactate dehydrogenase (LDH), platelets, and D-dimer and ECG [9, 43, 46].

The sporozoite infection model with infectious mosquitoes mimics the natural route of infection and allows the occurrence of natural and artificial conditions impacting the development of natural malaria infection, such as naturally or vaccine-induced immunity and drugs. The model has been used in the development and early clinical validation of vaccines [21, 24] and more recently has been established as a translational model for prophylactic drug development [5, 14–17]. Historically, the reliability of the model to support the development of drugs or vaccines has been impaired by the complexity and reproducibility of infective bites by mosquitoes; uncontrolled number of sporozoites inoculated by biting mosquitoes were limiting the interpretation of results from experimental challenge models [7]. The conduct of mosquito-challenge trials was complicated by the need for a mosquito insectary and entomological expertise and risk of adventitious infections [45, 52]. The recent availability of aseptically manufactured PfSPZ Challenge has changed the landscape for CHMI trials with sporozoites and expanded the global capacity to conduct CHMI trials [86], particularly in endemic countries. It also allowed establishing standardized CHMI conditions for drug development [27, 68].

The development of cryopreserved sporozoites helped to produce large scale vaccination in endemic countries following the initial observation that irradiated sporozoites could induce fully protective immune response [87]. With the industrial success of Sanaria Inc to produce large quantity of cryopreserved sporozoites, several CHMI studies have been conducted in malaria endemic countries in Africa to prepare the ground for vaccination campaigns [9, 12, 27, 67, 72–77]. These studies have demonstrated the feasibility of CHMI studies in Africa and allowed to assess the influence of pre-established immunity and malaria related genetic factors such as G6PD and sickle cell trait in the readout of the results [72]. This study indicated that naturally adaptive immunity to malaria and sickle cell trait prolonged prepatent time. The African CHMI studies have also demonstrated the need for addressing the technical and scientific as well as ethical and regulatory challenges [8, 12, 88]. These include prior consultation with key stakeholders, community sensitization, extensive exclusion criteria, consenting issues, challenges of screening of previous exposure, screening asymptomatic parasitemia, confounding of haemoglobinopathies and natural immunity, clinical & laboratory capacity and multitiered system of ethical review.

This systematic review lacks meta-analysis of data on CHMI trials conducted in healthy malaria naïve population and those in endemic countries due to heterogeneous design of trials conducted. However, the data clearly emphasize that these types of experiments can now be conducted in a reliable and systematic manner in well-equipped clinical settings in Africa or other endemic countries. It also provides a comprehensive qualitative evidence on reliability of sporozoite challenge for testing novel therapeutic and prophylactic interventions in healthy volunteers and guide further clinical development. Altogether, the data advocate for further capacity building initiatives to malaria-endemic country based clinical research centers.

## Conclusion and future perspectives

Current evidence indicates that mosquito-based sporozoite challenge of healthy volunteers has been validated for several strains of *P. falciparum,* with five infective mosquito bites being the optimal dose resulting in reproducible infectivity, prepatent period and safety profiles. Mosquito-based sporozoite challenge is a gold standard as it mimics natural infection, however, the method is not yet evaluated in the African setting.

The consistent safety and infectivity of PfSPZ challenge is verified among populations in Europe, America, and Africa. Infective doses are optimized, with DVI of PfSPZ challenge being a new emerging gold standard, including in malaria endemic African population, although there was variability in infection outcomes. Both methods of sporozoite challenge are well characterized for consistent safety profiles. The *P. vivax* CHMI model using infected mosquitoes has also been validated in few studies, with some limitations including lack of optimization of long-term culture and CHMI procedures.

Sporozoite challenge model is extensively used for testing vaccine efficacy, although few vaccines have been advanced to date. The applicability of the method is also verified in a few prophylactic drug efficacy studies. However, for efficacy studies in African population, PfSPZ challenge is recommended based on current evidence on safety, infectivity and ease of logistical requirement.

The understanding of sporozoite CHMI is advancing, however, gaps in current scientific knowledge to be addressed by future CHMI studies include further assessment of genetic and antigenic diversity of *P. falciparum*, use of bolus dose of sporozoites in CHMI compared to multiple exposures occurring naturally, comparative studies on wild and lab-cultivated parasites and mosquito vectors, CHMI-mosquito studies in malaria endemic settings in Africa, impact of natural immunity and hemoglobinopathies on CHMI outcomes, development of long-term *in-vitro* culture of *P. vivax* and cryopreserved *Plasmodium vivax* sporozoites (PvSPZ).

## Data Availability

The datasets used and/or analyzed during the current study are available from the corresponding author on reasonable request.

## Abbreviations

CHMI: Controlled human malaria infection
PfSPZ: Plasmodium falciparum sporozoite
PvSPZ: Plasmodium vivax sporozoite
P.falciparum: Plasmodium falciparum
P. vivax: Plasmodium vivax
RCT: Randomized controlled trial
TBS: Thick blood smears
CPS: Chemoprophylaxis
qPCR: Quantitative polymerase chain reaction
G6PD: Glucose -6-phosphate dehydrogenase
DVI: Direct venous inoculation
ALT: Alanine aminotransferase
AST: Aspartate aminotransferase
ID: Intraderma
IM: Intramuscular
SAE: Serious adverse event
